# Recovering tissue volumes from a single bioimpedance spectrum: an error analysis from phantoms and simulation

**DOI:** 10.2478/joeb-2026-0019

**Published:** 2026-11-09

**Authors:** Gautam Anand, Anubha Kalra, Arunima Ballav, Rebacca Sara Mani

**Affiliations:** Department of Electrical and Electronic Engineering, School of Engineering Computer and Mathematical Sciences, Auckland University of Technology, Auckland, New Zealand; AUT Institute of Biomedical Technologies, Auckland University of Technology, Auckland, New Zealand

**Keywords:** bioimpedance spectroscopy, body composition, finite element modelling, tissue-mimicking phantoms, electrode configuration, stray capacitance

## Abstract

Bioelectrical impedance analysis (BIA) estimates body composition through equations calibrated on reference populations, and its error for an individual is typically about ±8 percentage points of body fat. A physics-based estimate from a single spectrum would remove this dependence on calibration, although the errors that limit such an estimate have not yet been quantified against a known truth. This work quantifies these errors with three-layer phantoms of known composition, a finite element (FE) model verified against Ansys Maxwell, and simulations with phantom and tissue properties over 1–251 kHz. Without fitted parameters, the FE model predicted the impedance of two cylindrical phantoms measured with spot electrodes to within 1.1% and 6%. With the measurement scale fixed from the phantom dimensions, the fat fraction of all three phantoms was recovered to within 3 percentage points. The fat-free and water fractions, whose material spectra were very similar, were poorly separated. Without a known scale, recovery failed for the two gelatine phantoms. The fat fraction of an agar phantom with a more distinct fat spectrum, however, was still recovered to within 1 percentage point. In simulations, tissue spectra allowed recovery without a known scale, with typical errors of 3–5 percentage points, but only when the tissue properties were known exactly. A 10–20% spread in these properties raised the typical errors to 7–15 percentage points, and an extension of the band to 2 MHz did not reduce them. An uncorrected setup error, measured as 120 pF with a 14 ns delay, reduced recovery of the tissue set from 97% to 58%. Physics-based recovery therefore requires either a known scale, together with a validated electrode model and a corrected setup, or material spectra distinct enough to fix the scale. These findings define the requirements for phantoms intended to test such methods.

## Introduction

Body composition, in terms of fat, fat-free tissue and body water, is closely linked with nutritional status, cardiometabolic risk and the progression of chronic disease [1,2]. Its assessment is therefore relevant across clinical nutrition, chronic disease management and population health. Bioelectrical impedance analysis (BIA) is one of the most accessible tools for this purpose, as it is non-invasive, inexpensive and quick to perform [1]. The technique relies on the difference in electrical conductivity between water-rich lean tissue and poorly conducting fat. Fat-free tissue also shows a frequency-dependent response, which arises from the capacitance of cell membranes [3,4].

Most BIA devices do not estimate composition from this physics directly. Instead, the measured impedance is combined with height, weight, age and sex in a regression equation. Such equations are calibrated against a reference method such as dual-energy X-ray absorptiometry (DXA) in a large population [1,5]. In validation studies against DXA, these devices show mean errors of about 2–4 percentage points in body fat. The 95% limits of agreement for individuals, however, span about 12–27 percentage points [6,7,8,9]. As such, the estimate is reliable for groups but considerably less so for an individual. Fig. 1 illustrates the range of true body fat that is consistent with a single reading.

**Fig. 1:**
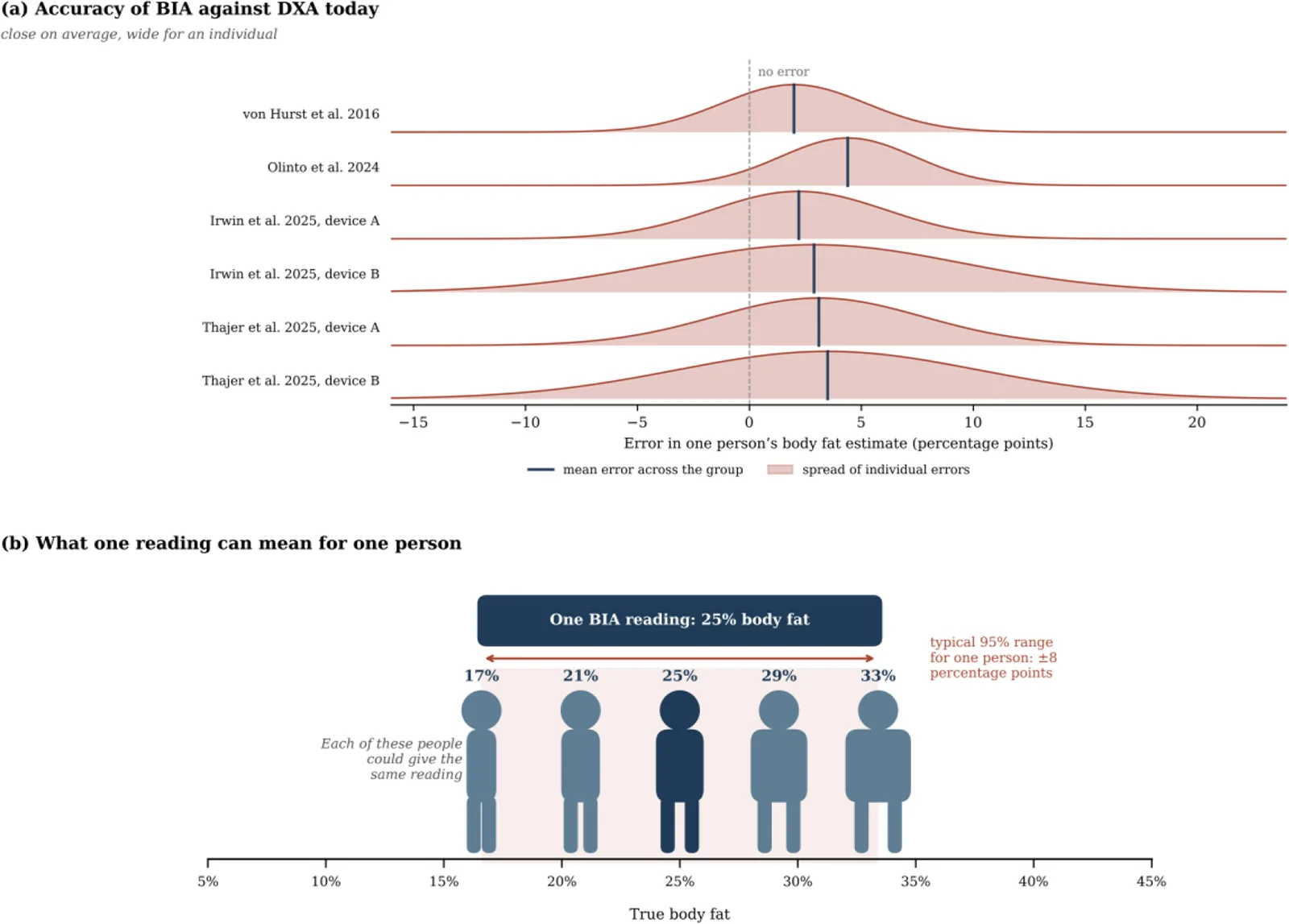
Individual error of BIA against DXA. (a) Distribution of individual errors in body fat, derived from the mean error and the 95% limits of agreement reported in four validation studies [6–9]. (b) Range of true body fat consistent with a single reading of 25%, based on the median half-width of these limits of about ±8 percentage points.

Bioimpedance spectroscopy (BIS) measures the impedance over a range of frequencies, and mixture models relate the measured spectrum to tissue volumes [5,10,11]. De Lorenzo et al. [12] applied Hanai mixture theory to estimate body water from fundamental principles. These estimates, however, still rely on resistivity coefficients and corrections for body mass index, which are calibrated on populations [13,14]. Gautier et al. [2] identified the nonuniform flow of current through the tissues as one of the main obstacles for such models. Hence, it remains unclear whether a single surface spectrum carries enough information to separate three tissue volumes.

Tissue-mimicking phantoms provide a direct means of testing this question, since their true composition is fixed by construction. Anand et al. [15] developed forearm phantoms that mimic the dielectric properties of fat, muscle and blood between 1 kHz and 349 kHz. Yu et al. [16] combined an Ansys model of the wrist with a gelatine phantom and showed that band electrodes produce a more uniform current than spot electrodes. Bublex et al. developed polyvinyl alcohol (PVA) phantoms of muscle [17] and fat [18] and later assembled them into a multilayer leg phantom [19]. Longhitano et al. [20] showed that Ansys Maxwell models of measuring cells closely reproduce the measured impedance of such materials. Table 1 summarises these and related works. Once a phantom spectrum is used to test a physical model, measurement errors become as important as the model itself. Electrode polarisation (EP) adds to the measured impedance in two-electrode measurements [21]. Grimnes and Martinsen [22] showed that four-electrode measurements are also affected by polarisation, separate current paths and common-mode signals. Stray capacitance distorts the spectrum at high frequencies, as modelled by Scharfetter et al. [23] and observed by Bolton et al. [24]. Buendia et al. [25] proposed a correction for this effect.

**Table 1:** Related works on phantoms, modelling and measurement errors in bioimpedance spectroscopy.

**Study**	**Focus**	**Frequency range**	**Relevance to this work**
Anand et al. [15]	Forearm phantom of fat, muscle and blood	1 kHz–349 kHz	Phantom materials and measurement device
Yu et al. [16]	Ansys model and wrist phantom	1 kHz–1 MHz	Electrode type controls how uniform the current is
Bublex et al. [17,18,19]	PVA phantoms of muscle and fat, multilayer leg	1 kHz–1 MHz	Alternative phantom materials and layer bonding
Longhitano et al. [20]	Ansys Maxwell model of measuring cells	1 kHz–1 MHz	Maxwell models match cell measurements
Grimnes and Martinsen [22]	Errors in four-electrode measurements	Not frequency-specific	Four-electrode systems are still affected by errors
Scharfetter et al. [23]	Model of stray capacitance errors	5 kHz–1 MHz	Cause of the high-frequency phase difference
Buendia et al. [25]	Correction of capacitive leakage	Not frequency-specific	Basis for the stray capacitance correction
Jaffrin and Morel [5]	Review of BIS and BIA methods	Multi-frequency	Current practice relies on calibration equations

A further difficulty is that a model can reproduce a measured spectrum closely while the fitted composition is wrong. This occurs when several different compositions produce almost the same spectrum, a property known as ill-conditioning [26]. Nejadgholi et al. [27] showed that even the four parameters of the Cole model can vary widely between sessions when the spectrum changes only slightly. Physics-informed neural networks have been proposed for related inverse problems in electrical impedance tomography [28,29]. These methods, however, remain limited by the information carried by the measurement itself, rather than by the search method alone.

The electrode arrangement and the thickness of surface layers are known to affect bioimpedance measurements [30,31,32]. The combined effect of the model, the materials and the measurement errors on the recovery of composition has, however, not been quantified against a known truth. This work aims at identifying what limits the recovery of the volumes of three tissue compartments from a single surface spectrum. Measured phantoms of two geometries were combined with an FE model and with three sets of material properties, ranging from phantom materials to real tissue. The phantoms test the model and the recovery directly, while the simulations extend the test to materials and errors that were not available as phantoms. In this way, the study separates the effect of the model from that of the materials, and it accounts for the measurement errors that limit the agreement between model and measurement.

The rest of this paper is organised as follows. The materials and methods section presents the phantoms, the measurements, the models and the recovery tests. The results section first establishes the accuracy of the model, then presents the measured phantoms and finally the simulations. The discussion examines these findings together with their limitations, and the paper closes with the conclusion.

## Materials and methods

### Overview of the workflow

The study followed two connected strands, which led to a single error analysis (Fig. 2). In the first strand, the materials and three phantoms of known composition were measured. These measurements were compared with an FE model of the actual electrodes and with a mixing model, with no fitted parameters, and the composition was then recovered from each measured spectrum. In the second strand, the same FE model was used to simulate spectra for phantom and tissue properties, with a generic layout of spot electrodes. Since the true composition of these spectra was known exactly, the recovery could be tested under controlled random noise and realistic errors. The findings of both strands were then combined into the error analysis.

**Fig. 2:**
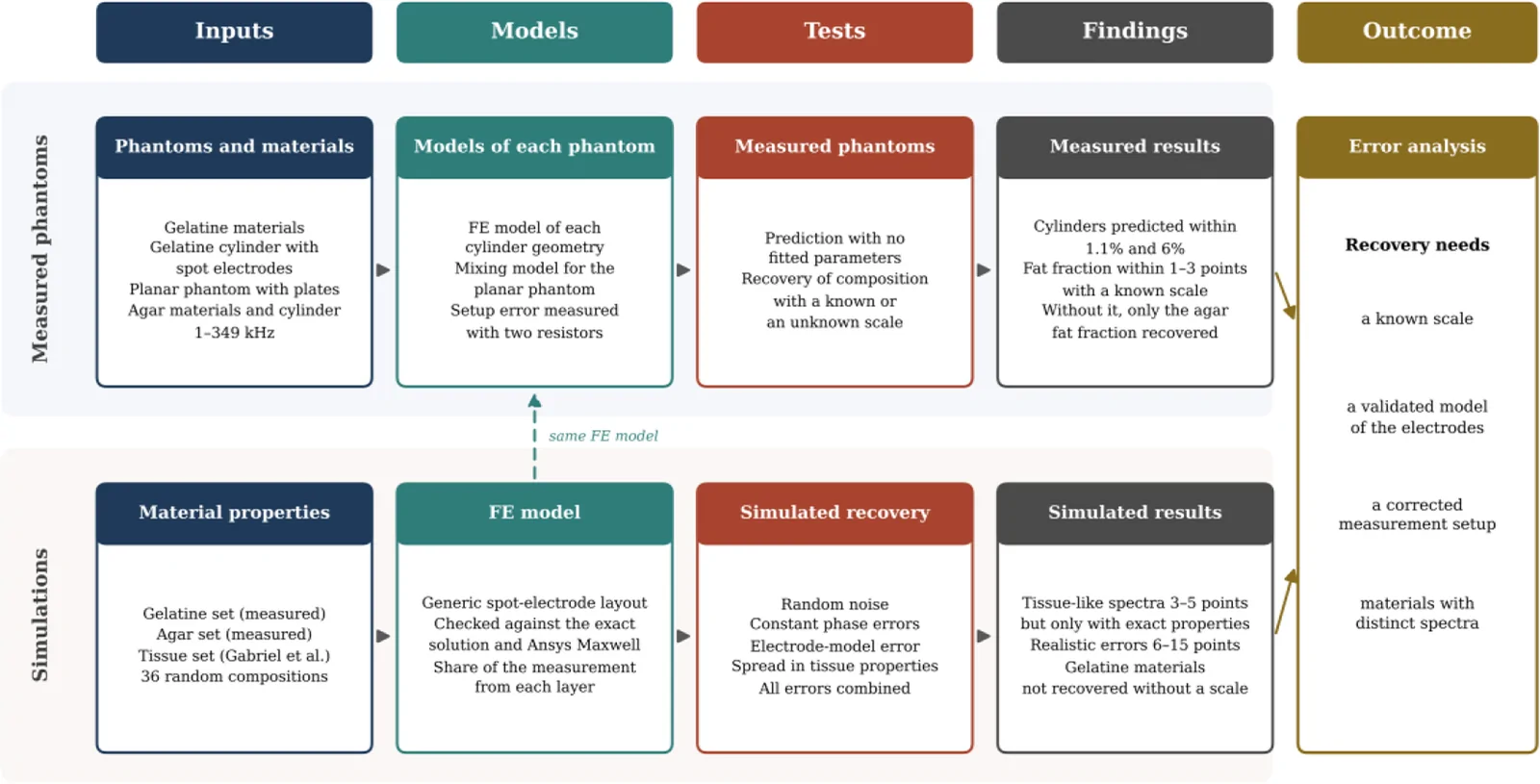
Workflow of the study. The upper strand follows the measured phantoms and the lower strand the simulations, and both lead to the error analysis. FE, finite element.

### Phantom materials and geometries

Three materials were used to represent the three compartments, namely fat mass (FM), fat-free mass (FFM) and total body water (TBW), following earlier phantom work by this group [15]. The TBW material consisted of 100 ml deionised water and 0.9 g sodium chloride (NaCl). The FFM material consisted of 100 ml deionised water, 10 g gelatine and 0.9 g NaCl. The FM material consisted of 100 ml deionised water and 10 g gelatine, with no added salt. These materials are referred to as the gelatine set. The same recipes were used for the separate measurements of each material and for the two gelatine phantoms, although each was made in a separate preparation.

A second set, referred to as the agar set, used the same TBW and FFM recipes. Its FM material consisted of 200 ml deionised water, 200 ml propylene glycol, 60 g agar and 20 g gelatine. This recipe was intended to bring the conductivity of FM closer to that of real fat. Each material of this set was also measured on its own, and the same recipes were used to build the agar cylinder.

Each material was measured on its own in a two-electrode cell with copper plates on opposite walls. For the gelatine set, the cell had a 5 cm electrode spacing and a 5 cm × 1 cm cross-section. For the agar set, the cell was calibrated with the same 0.9% saline used for TBW.

Both cylinders were built in the same tube as concentric layers, with TBW at the centre, FFM in the middle and FM on the outside (Table 2). The tube was 20 cm long and had an outer radius of 3.6 cm. Its wall was at most 2 mm thick, so the gel had a radius of about 3.4 cm. Four spot electrodes, 2 cm in diameter, were fitted into slots in a line along the curved surface. The two voltage electrodes were 6 cm apart and the two current electrodes 11.5 cm apart, measured centre to centre with an uncertainty of about 3 mm. The planar phantom consisted of three stacked layers in the measurement cell, with a total height of 0.8 cm. Its composition was set by the volumes of the three materials poured into the cell.

**Table 2:** Geometry, electrodes and composition by volume of each phantom.

**Phantom**	**Geometry**	**Electrodes**	**FM (%)**	**FFM (%)**	**TBW (%)**
Cylinder, gelatine set	Concentric layers, gel radius 3.4 cm, length 20 cm	Four spot electrodes, voltage 6 cm and current 11.5 cm apart	46.2	42.7	11.1
Planar, gelatine set	Stacked layers, height 0.8 cm	Two-electrode plates	31.3	28.9	39.8
Cylinder, agar set	Concentric layers, gel radius 3.4 cm, length 20 cm	Four spot electrodes, as above	50.3	44.7	5.0

### Measurements

All measurements were made with a Quadra impedance spectroscopy system (Eliko, Tallinn, Estonia) at 15 frequencies between 1 kHz and 349 kHz. Each material and each phantom was measured about 4 hours after it was made, in a room held at about 22 °C by air conditioning. The temperature was not logged. The materials of the gelatine set were measured in March 2026 and the gelatine phantoms in May 2026. The materials of the agar set were measured in July 2026 and the agar cylinder in October 2026. Each material and each gelatine phantom was measured in three consecutive tests, and the agar cylinder in 1,175 consecutive sweeps. Each spectrum was taken as the mean of its repeated sweeps. Within each session, the coefficient of variation of the magnitude was at most 0.25%, and the standard deviation of the phase was at most 0.02°. The 349 kHz point was excluded from all fits, since every spectrum showed a drop at this frequency that originates in the instrument.

### Mixing model

The stacked layers of the planar phantom each span the full gap between its electrodes, so each layer acts as a separate conducting path. The planar phantom was therefore described with a parallel mixing model [10,11],

(1) $$Y_{\mathrm{composite}}\left(f\right)=C\sum_{i}\phi_{i}\;\;Y_{i}\left(f\right),\;\;\;\;\;\;\;\;\;\;\;\;\;\;\sum_{i}\phi_{i}=1,$$

where *Y_i_* is the measured admittance of each material, *φ_i_* is its volume fraction and *C* is a scale factor. For a uniform conductor, *C* follows directly from the dimensions,

(2) $$C=\frac{A_{\mathrm{phantom}}/L_{\mathrm{phantom}}}{A_{\mathrm{cell}}/L_{\mathrm{cell}}},$$

where *A* is the cross-sectional area and *L* is the length of the current path. The scale factor can therefore be fixed from measured dimensions instead of being fitted. In the cylinders, the current from spot electrodes does not flow evenly through the layers, and these were described with the FE model below.

### Finite element model

The mixing model assumes that current flows uniformly along the phantom, which holds only when current enters through plates covering the full ends. An FE model was therefore developed for other electrode arrangements. The electric potential *V* in the phantom satisfies

(3) $$\nabla \cdot \left(\sigma^{*}\nabla V\right)=0,\;\;\;\;\;\;\;\;\;\;\sigma^{*}=\sigma +j\omega \varepsilon_{0}\varepsilon_{r},$$

where σ^*^ is the complex conductivity of each layer, *ω* is the angular frequency and *ε*_0_ is the permittivity of free space. The frequencies used here are low enough for the problem to be treated as quasi-static [33], so the magnetic field and wave effects were neglected.

The FE model was written in Python 3.12 with the open-source scikit-fem library, version 12.0.2 [34], together with NumPy and SciPy. The layers of the phantom are round, whereas spot electrodes cover only a small part of its surface. The potential was therefore written as a sum of azimuthal modes, *V* = ∑*_m_ V_m_* (*r*, *z*)cos(*mθ*), where *r* is the radius, *z* is the position along the phantom and *θ* is the angle around it. Each three-dimensional problem was thereby reduced to a set of two-dimensional problems in *r* and *z*, which gives the full three-dimensional result for round layers at a much lower computational cost.

Each mode was solved on a structured mesh of four-node elements with about 3,000 nodes. The mesh was refined near the outer surface, at the boundaries between layers and around the electrodes, where the current changes most rapidly. The outer surface was insulated except under the electrodes. Each current electrode was represented as a uniform current over its area, and each voltage electrode recorded the average potential over its area. The resulting equations were solved with the sparse direct solver of SciPy. One geometry at one frequency was solved in about one second on a desktop computer.

Three electrode arrangements were modelled. The first used plates at both ends, for which the mixing model is exact. The second reproduced the two measured cylinders, with the geometry given above. The third was a generic layout used in the simulations (Fig. 3). It had four spot electrodes 1 cm in diameter, placed in a line with a 3 cm spacing on a cylinder 10 cm long.

**Fig. 3:**
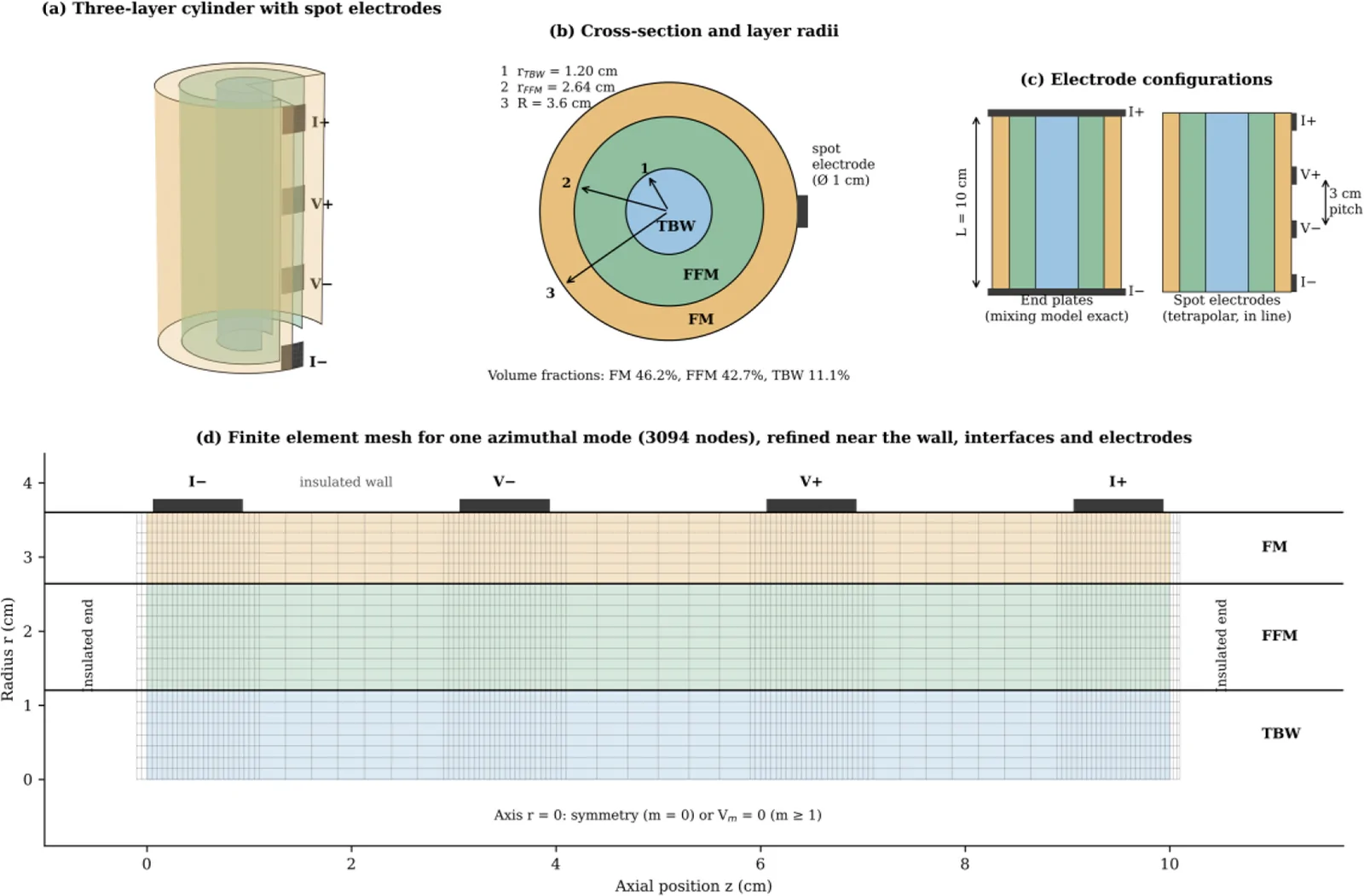
Generic simulation setup. (a) Cut-away view of the three-layer cylinder with four spot electrodes. (b) Cross-section with the layer radii. (c) End plates and spot electrodes with their dimensions. (d) Finite element mesh for one azimuthal mode, with the boundary conditions and the electrode positions.

### Model verification

The FE model was verified in three ways. For end plates, its result was compared with the exact solution of the mixing model. The numerical settings were then checked by refining the mesh, by increasing the number of modes and by exchanging the current and voltage electrodes, which should give the same result. For spot electrodes, the FE model was compared with an independent model of the generic layout in Ansys Maxwell 3D (Ansys Inc., Canonsburg, PA, USA), filled with the gelatine set. In Maxwell, the electrodes were copper discs shaped to the curved surface of the phantom. A voltage of 1 V was applied between the outer electrodes, and the voltage between the inner electrodes was recorded. Since Maxwell does not accept relative permittivities below one, such values were set to one in both models for this comparison.

The share of the measurement that arises from each layer was also computed. This share, or sensitivity, follows from the lead field theory of Geselowitz [35] and was defined as

(4) Si=−σiReZ∂ReZ∂σi,

which was obtained by increasing the conductivity of each layer by 1%. Only conductivity was varied, since it dominates the complex conductivity of all three materials in this band. The three shares add up to one, and for end plates each share equals the share of that layer in the total conductance.

### Material sets

Three sets of material properties were compared. The gelatine and agar sets were taken from the measurements of the individual materials. The tissue set represented real fat, muscle and blood, computed from the model of Gabriel et al. [4] as given in the IFAC-CNR database [36]. The tissue set is a three-tissue model, since blood is not equivalent to TBW, which in the body is largely contained within lean tissue. The results for the tissue set therefore describe three separate tissue volumes rather than body composition in the clinical sense. Some measured phases of the phantom materials were slightly positive at higher frequencies, which corresponds to a negative permittivity. Positive phase is a known artefact of measurement setups [37,38], and these values were retained as measured.

### Measurement error models

Electrode polarisation was estimated from the TBW material. A simple salt solution has no internal structure that could cause a frequency-dependent response, so any such response can be attributed to the electrodes. The polarisation was described as

(5) $$Z\left(f\right)=R+\frac{1}{Q{\left(j\omega \right)}^{n}},$$

and this term was subtracted from all three materials measured in the same cell. This assumes that the polarisation of FFM and FM equals that of TBW. FFM and TBW contain the same amount of salt, and the impedance of FM is at least eight times larger than that of the other two materials. To test whether the conclusions depend on this assumption, the fits of the measured cylinders were repeated with the term removed, tripled for FM, and halved or doubled for FFM. The setup error of the measurement connection was described as a stray capacitance *C_p_* across the sample together with a fixed delay *τ* [23,24,25],

(6) $$Z_{\mathrm{meas}}=\frac{Ze^{-j\omega \tau }}{1+j\omega C_{p}Z},$$

which produces a phase error that grows with frequency. Both parameters were measured with precision resistors of 27 Ω and 100 Ω connected in place of the gelatine cylinder with the same leads. For a resistor *R*, equation (6) gives a phase of about −*ω*(*τ* + *C_p_R*). The phase of both resistors was proportional to frequency. At 251 kHz, it was about −1.6° for 27 Ω and 1.5 times larger for 100 Ω. These two values give *C_p_* = 121 pF and *τ* = 14.4 ns. The resulting correction was applied to the measured spectrum without any fitting.

### Recovery from the measured phantoms

The composition of each phantom was recovered directly from its measured spectrum, with one fit for each phantom. The planar phantom was fitted with the mixing model. Each cylinder was fitted with the FE model of its geometry, using spectra computed for 66 compositions and interpolated between them, with each fraction limited to between 4% and 92%. For each value of TBW, in steps of 0.2%, the fit found the best FM by a continuous search, and the composition with the lowest residual was taken as the result. The scale factor was either fixed from the dimensions or left free.

For each value of one fraction, the fit was also repeated with the other fractions free, which gives a residual profile for that fraction. The range of each fraction whose fit came within 1% of the lowest residual was recorded, as a measure of how well the spectrum fixed that fraction. The spectrum of the gelatine cylinder was first corrected for the setup error of its connection. The agar cylinder was fitted as measured, and the effect of the same correction on its phase was also examined. Each fit was repeated with the scale changed by ±5%, which reflects the uncertainty in the electrode positions and a difference in temperature of up to about 2.5 °C. For a known scale, the ranges were combined over these three fits. None of these fits had any parameter other than the composition and, where stated, the scale.

### Simulated recovery tests

The simulations extend the recovery test to tissue-like materials, which were not available as phantoms, and to a wider range of compositions and errors. Thirty-six random compositions were generated, each with every fraction at about 4% or more. For each composition, the spectrum was computed with the mixing model and with the FE model of the generic spot-electrode layout. Random noise was then added, with a standard deviation of 0.1% or 1% in magnitude and 0.01° or 0.1° in phase, and each case was repeated ten times.

Each spectrum was fitted with the mixing model and with the FE model. For the FE model, spectra were computed for 66 compositions spread evenly over all possible mixtures, and the model interpolated between them. The scale factor was either known or left free, and every fit searched all compositions in steps of 0.2%. Unless stated otherwise, all simulated spectra and fits covered the same band of 1–251 kHz as the measurements. For the tissue set, the main tests were repeated with six additional frequencies, which extended the band to 500 kHz, 1 MHz and 2 MHz. The same 36 compositions and the same random noise were used in every test, so that the results of different tests can be compared directly.

Four further tests examined the effect of realistic errors. In the first, an error that is the same at all frequencies was added, namely a constant phase error of ±0.1° or ±0.3°. The same test was made with the uncorrected setup error measured on the resistors, a capacitance of 120 pF with a delay of 14 ns. In the second, the magnitude was changed by 17%, which corresponds to the difference between the FE model and Ansys Maxwell, while the fit assumed a known scale. In the third, the properties of real tissue were varied randomly by 5%, 10% or 20% around the values of Gabriel et al. [4], while the fit used the nominal values. In the fourth, the FE model was used to fit the spectrum computed by Ansys Maxwell, which tests whether recovery survives a change of model.

### Similarity of the material spectra

When the spectra of two materials have almost the same shape, a small measurement error can produce a large error in the recovered composition. This effect was summarised by an error amplification factor, defined as the largest factor by which a small error in the measured spectrum can grow in the recovered composition [26]. It was computed from a matrix with one column for each material. Each column held the real parts and then the imaginary parts of the complex conductivity at the 14 frequencies. Each column was scaled to unit length, and no further weighting was applied. The factor is the ratio of the largest to the smallest singular value of this matrix. Since each column is scaled separately, the factor describes the mixing model of equation (1) with an unknown scale. The same ratio was also computed for the FE model of the generic layout. For this purpose, the change of the logarithm of the magnitude and of the phase with each fraction was computed at the 36 test compositions. Each frequency was weighted by the noise of the simulations. A factor close to one indicates a well-conditioned problem.

### Statistical analysis

Agreement between a model and a measurement was expressed as the mean difference in magnitude and the root-mean-square difference in phase. The accuracy of recovery was expressed as the typical error of each fraction, given as the root-mean-square difference from the true value in percentage points. A composition was counted as recovered when all three fractions were within 15 percentage points of the truth, and the recovery rate is the percentage of compositions recovered. Recovery rates from the simulations were reported with 95% confidence intervals across the 36 compositions.

## Results

### Model verification

For end plates, the FE model matched the exact mixing model at every frequency, with a largest difference below 0.00001% (Fig. 4). The result for spot electrodes did not change beyond 20 modes. A mesh with about four times as many nodes changed the magnitude by 0.4% and the shape of the spectrum by less than 0.01%. An exchange of the current and voltage electrodes changed the result by less than 0.001%.

**Fig. 4:**
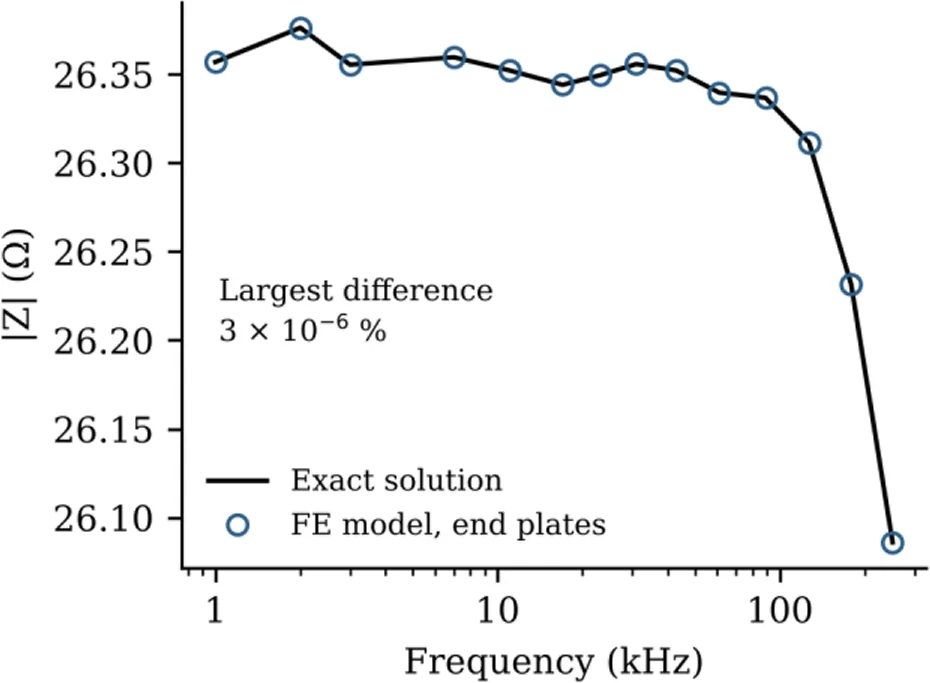
Verification of the FE model with end plates. The impedance magnitude of the gelatine cylinder composition from the FE model is compared with the exact solution of the mixing model.

### Current path with end plates and spot electrodes

Fig. 5 compares the current flow for end plates and spot electrodes. With end plates, the current flowed straight and evenly through every layer. With spot electrodes, the current entered and left through the outer FM layer and spread inwards before flowing along the inner layers. As a result, the share of the measurement arising from FM increased from 9% to 23% for the gelatine set and from 5% to 32% for the tissue set.

**Fig. 5:**
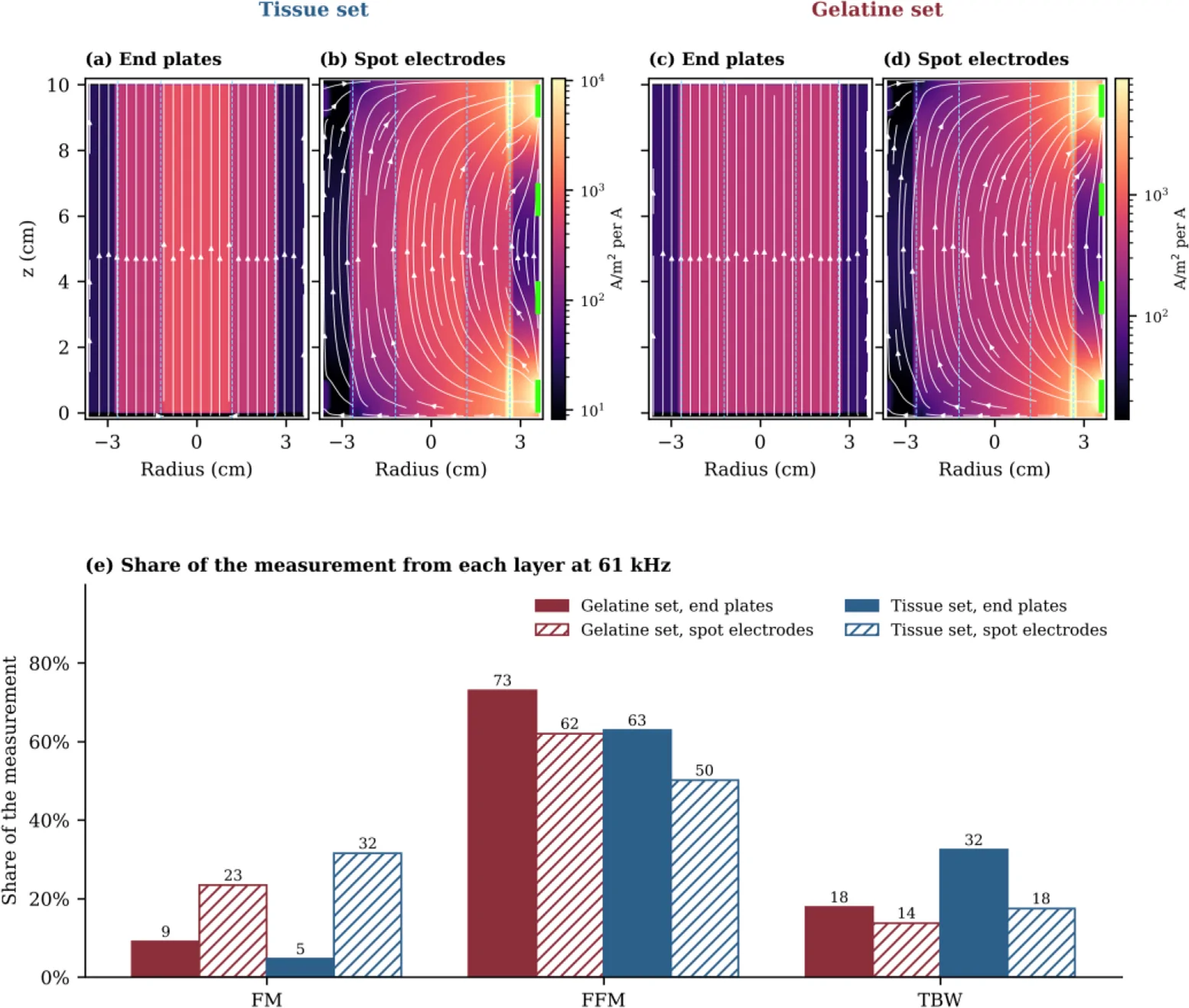
Current flow at 61 kHz in the generic cylinder. (a, c) End plates. (b, d) Spot electrodes on the right-hand side. Panels (a) and (b) use the tissue set and panels (c) and (d) the gelatine set, and each pair shares one colour scale. Colour shows the current density for 1 A injected, white lines follow the current, dashed lines mark the layer boundaries and green marks show the spot electrodes. (e) Share of the measurement arising from each layer.

### Comparison with Ansys Maxwell

Fig. 6 compares the FE model with Ansys Maxwell for the generic layout with the composition of the gelatine cylinder. The magnitude from Maxwell was 17.2% higher at every frequency, and this difference was constant across the band (Fig. 6a). After both spectra were divided by their value at 1 kHz, they agreed to within 0.02 percentage points (Fig. 6b), and the phase agreed to within 0.1° (Fig. 6c). Maxwell gave an FM share of 16%, against 23% from the FE model and 9% for end plates (Fig. 6d).

**Fig. 6:**
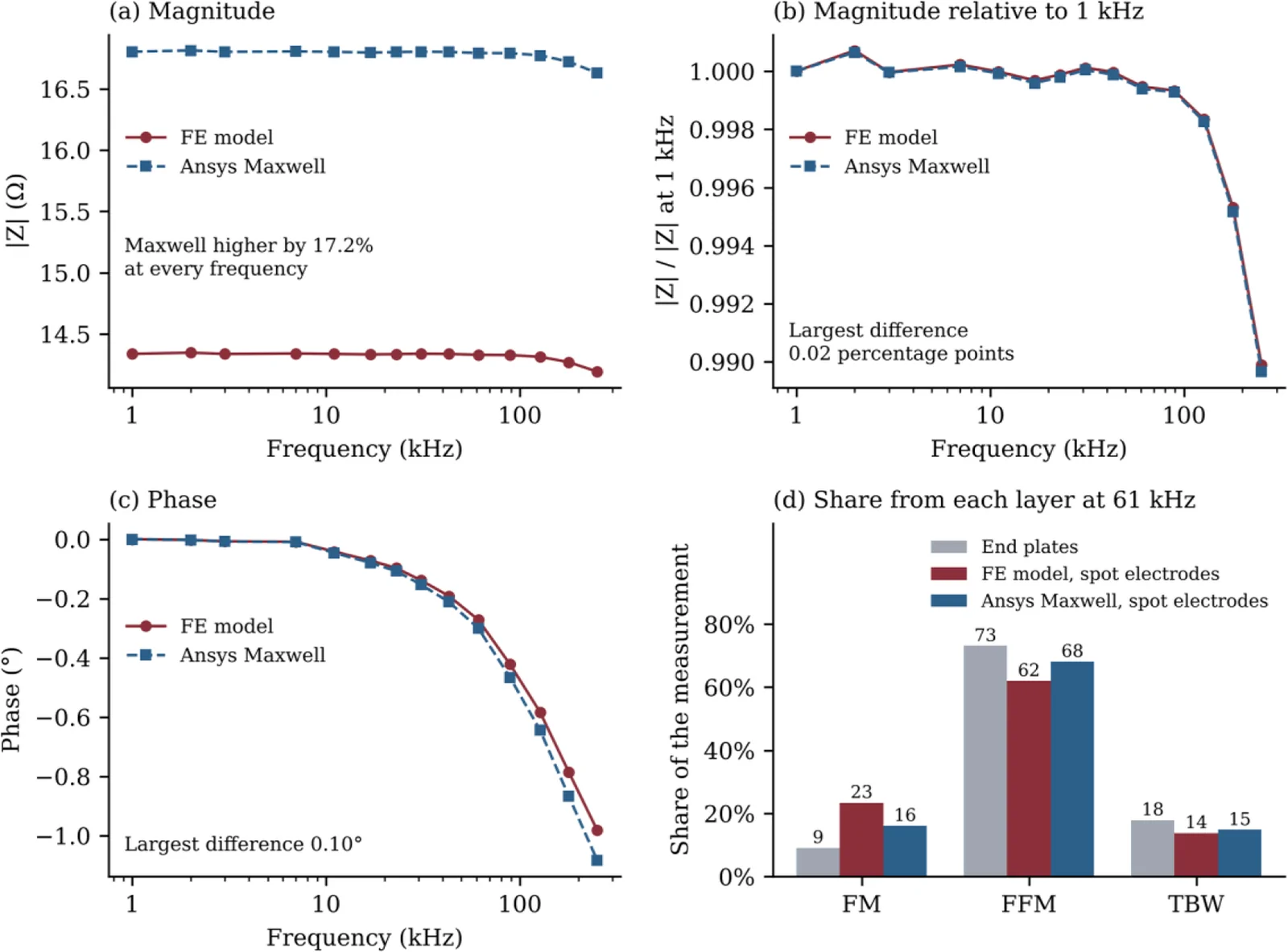
Comparison of the FE model with Ansys Maxwell for the generic spot-electrode layout with the composition of the gelatine cylinder. (a) Impedance magnitude. (b) Magnitude relative to its value at 1 kHz. (c) Phase. (d) Share of the measurement arising from each layer at 61 kHz.

When the Maxwell spectrum was fitted with the FE model, FM was overestimated. With the scale factor left free, the fit gave 62% FM, 34% FFM and 4% TBW, against a true 46%, 43% and 11%. With the scale factor taken from the FE model, the fit gave 52%, 43% and 6%, so the higher magnitude from Maxwell was taken up mainly by FM.

### Comparison with the measured phantoms

With no fitted parameters, the FE model of the measured geometry predicted 26.04 Ω for the gelatine cylinder at 1 kHz, against 26.33 Ω measured (Fig. 7a). The mean difference over the band was 1.1%. An uncertainty of 3 mm in the spacing of the current electrodes changes this prediction by up to about 7%. The mixing model over the section between the voltage electrodes predicted only 17.7 Ω, since the current in this section was not uniform. The phase of the FE model, however, differed from the measured phase at the upper frequencies (Fig. 7b). For the planar phantom, the mixing model matched the measurement with a mean error of 0.6% when the scale factor was fitted.

**Fig. 7:**
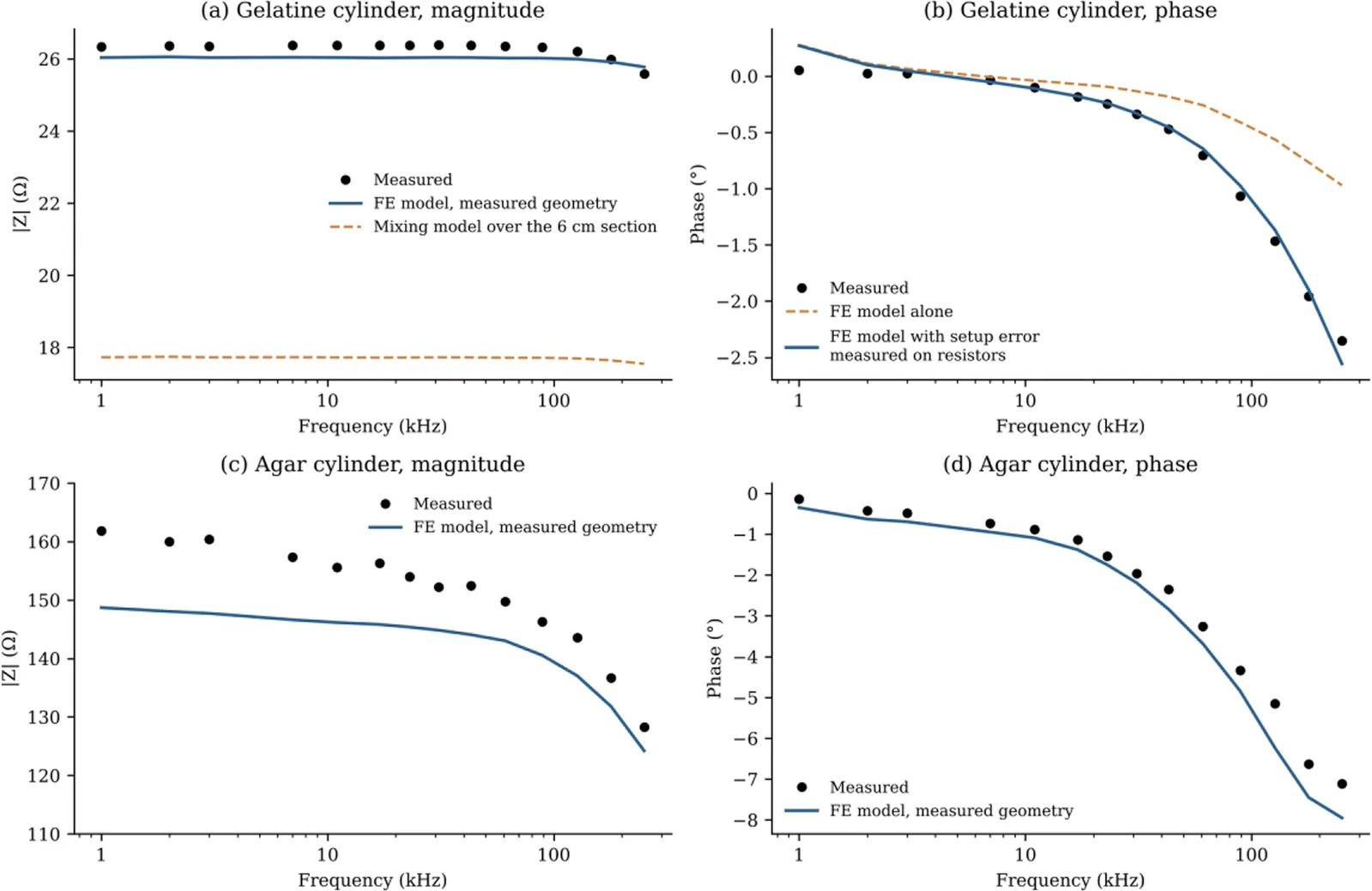
Measured cylinders compared with the FE model of their geometry, with no fitted parameters. (a) Magnitude of the gelatine cylinder, together with the mixing model over the section between the voltage electrodes. (b) Phase of the gelatine cylinder from the FE model alone and with the setup error measured on precision resistors. (c) Magnitude and (d) phase of the agar cylinder.

For the agar cylinder, the FE model predicted 149 Ω at 1 kHz, against 162 Ω measured (Fig. 7c). The measurement was higher by 6.0% on average, and this difference fell from about 9% at 1 kHz to about 3% at 251 kHz. The phase agreed to within 0.51° without any correction (Fig. 7d). The setup correction measured for the gelatine cylinder increased this difference to 1.53°, and it was therefore not applied to the agar cylinder.

### Correction of the phase difference

Fig. 8 summarises the phase correction. For the gelatine cylinder, the FE model with the polarisation-corrected materials differed from the measured phase by 0.60°, and this difference grew towards the upper end of the band. To identify its source, two precision resistors of 27 Ω and 100 Ω were measured in place of the cylinder with the same connection. Their phase revealed a setup error consisting of a capacitance of about 120 pF across the sample and a fixed delay of about 14 ns. When this measured setup error was applied to the FE model without any fitting, the phase difference fell to 0.09° (Fig. 8a). The polarisation correction made no difference to this result. For the planar phantom, a stray capacitance did not reduce the difference, whereas an additional polarisation term reduced it from 0.57° to 0.19° (Fig. 8b).

**Fig. 8:**
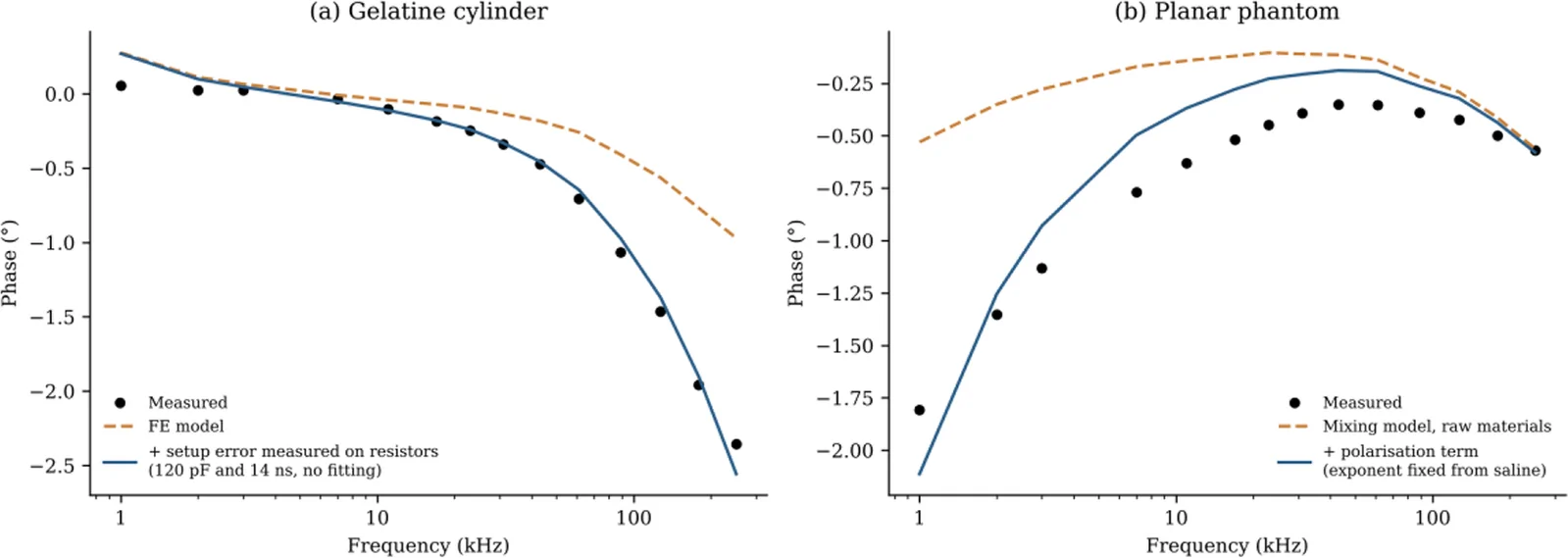
Correction of the phase difference between model and measurement. (a) Gelatine cylinder, with the setup error measured on precision resistors. (b) Planar phantom, with an additional polarisation term.

### Similarity of the material spectra

Fig. 9 compares the three material sets. The FM material of the agar set had a conductivity of 0.009 S/m at 1 kHz. This value is close to 0.022 S/m for real fat and much lower than 0.183 S/m for the gelatine set. Muscle and blood show a rising phase towards the upper end of the band, whereas the phantom materials remain nearly flat. The error amplification factor was 1,361 for the gelatine set, 2,356 for the agar set and 61 for the tissue set. For the FE model with spot electrodes and an unknown scale, the corresponding ratio was 813 for the gelatine set and 84 for the tissue set.

**Fig. 9:**
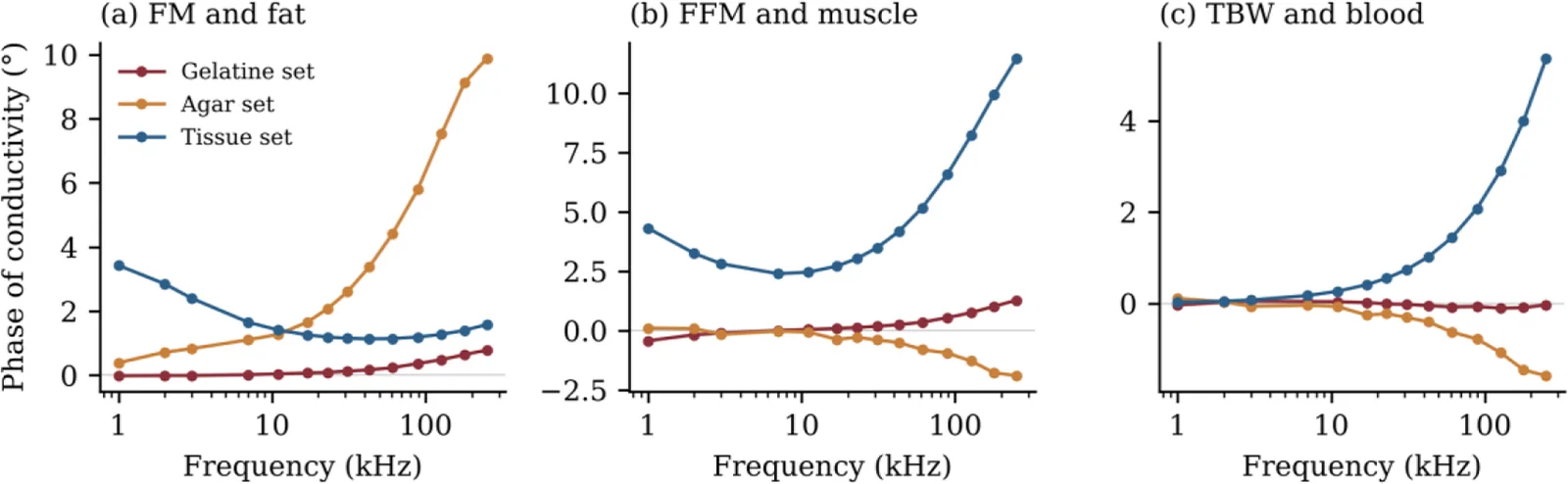
Phase of the complex conductivity of the FM, FFM and TBW materials for the gelatine, agar and tissue sets.

The spot electrodes therefore lowered the factor, but the gelatine set remained about ten times worse conditioned than the tissue set. The least well-determined combination involved mainly FM in the gelatine set, the split between FFM and TBW in the agar set, and FM against TBW in the tissue set.

### Recovery from the measured phantoms

Table 3 gives the composition recovered from each measured phantom, together with the range of each fraction over all fits within 1% of the lowest residual. With the scale fixed from the dimensions, FM was recovered to within 1 percentage point for both cylinders and within 3 percentage points for the planar phantom. With a change of 5% in the scale included, the range of FM was 45–49% for the gelatine cylinder, 50–52% for the agar cylinder and 23–33% for the planar phantom. For the gelatine cylinder, FFM was overestimated by 6 percentage points and TBW was found at 4%, the lowest value allowed by the search. Its ranges of FFM and TBW were narrow but did not contain the true values. For the agar cylinder and the planar phantom, FFM and TBW were 6–12 percentage points from the truth. Their fits, however, were almost equally good over wide ranges, up to 40 percentage points for the agar cylinder and about 20 percentage points for the planar phantom. The residual of each fit showed the same difference between the fractions. A change of 1 percentage point in FM from its best value raised the residual by 90% for the gelatine cylinder and by 280% for the agar cylinder. For the planar phantom, a change of 5 percentage points in FM raised it by 370%. A change of 10 percentage points in FFM or TBW raised the residual by about 20% for the gelatine cylinder. For the agar cylinder and the planar phantom, it raised the residual by less than 2%.

**Table 3:** Composition recovered from each measured phantom, with one fit of its measured spectrum. Values in brackets give the range of each fraction over all fits within 1% of the lowest residual. For a known scale, this range also includes a change of ±5% in the scale. The gelatine cylinder was corrected for the setup error, and no fit had a free parameter other than the composition and, where stated, the scale. The ranges show how well the spectrum fixes each fraction, but they do not include errors of the model.

**Phantom**	**Fit**	**FM (%)**	**FFM (%)**	**TBW (%)**
Gelatine cylinder	True composition	46.2	42.7	11.1
Gelatine cylinder	FE model, known scale	47 (45–49)	49 (46–51)	4 (4–5)
Gelatine cylinder	FE model, free scale	39 (36–39)	57 (57–59)	4 (4–5)
Agar cylinder	True composition	50.3	44.7	5.0
Agar cylinder	FE model, known scale	51 (50–52)	32 (6–46)	16 (4–43)
Agar cylinder	FE model, free scale	50 (50–54)	46 (4–46)	4 (4–44)
Planar	True composition	31.3	28.9	39.8
Planar	Mixing model, known scale	28 (23–33)	37 (28–48)	34 (24–45)
Planar	Mixing model, free scale	0 (0–6)	53 (42–62)	47 (36–58)

With the scale factor left free, the fit failed for both gelatine phantoms, giving 39% FM for the gelatine cylinder and 0% FM for the planar phantom. Without the setup correction, the gelatine cylinder gave 92% FFM. For the agar cylinder, the fit gave 50% FM, 46% FFM and 4% TBW, within 2 percentage points of the truth. The fitted scale was 8.8% above the nominal value, close to the 6% difference in magnitude shown in Fig. 7c. FM remained between 50% and 54% in all fits within 1% of the lowest residual. TBW in these fits, however, ranged from 4% to 44%, so the close agreement of FFM and TBW with the truth was not determined by the spectrum.

The polarisation correction of the materials had little effect on FM. When its term was removed, tripled for FM, or halved or doubled for FFM, the recovered FM of both cylinders changed by less than 0.3 percentage points with a known scale. The split between FFM and TBW of the gelatine cylinder moved by up to 6 percentage points. With a free scale, the gelatine cylinder gave between 4% and 82% FM across these cases, whereas the agar cylinder gave 50% in every case.

### Simulated recovery

Table 4 and Fig. 10 give the recovery for simulated spectra with random noise. With the mixing model, 33% of compositions were recovered for the gelatine set and 41% for the tissue set at 0.1% noise. The error in FM depended on how the electrodes were represented. With the FE model, an unknown scale factor and 1% noise, 85% of compositions were recovered for the tissue set and 30% for the gelatine set. The typical errors were 10, 5 and 7 percentage points for FM, FFM and TBW in the tissue set, against 29, 18 and 17 in the gelatine set. For the agar set, which was fitted with the mixing model only, recovery was 11% at 0.1% noise.

**Table 4:** Recovery rate for simulated spectra from the FE model with spot electrodes and random noise. The recovery rate is the percentage of compositions with all three fractions within 15 percentage points of the truth.

**Material set**	**Fitted with**	**Scale factor**	**0.1% noise (%)**	**1% noise (%)**
Gelatine	Mixing model	Unknown	33	12
Gelatine	FE model	Unknown	79	30
Gelatine	FE model	Known	100	91
Agar	Mixing model	Unknown	11	4
Tissue	Mixing model	Unknown	41	19
Tissue	FE model	Unknown	97	85
Tissue	FE model	Known	100	100

**Fig. 10:**
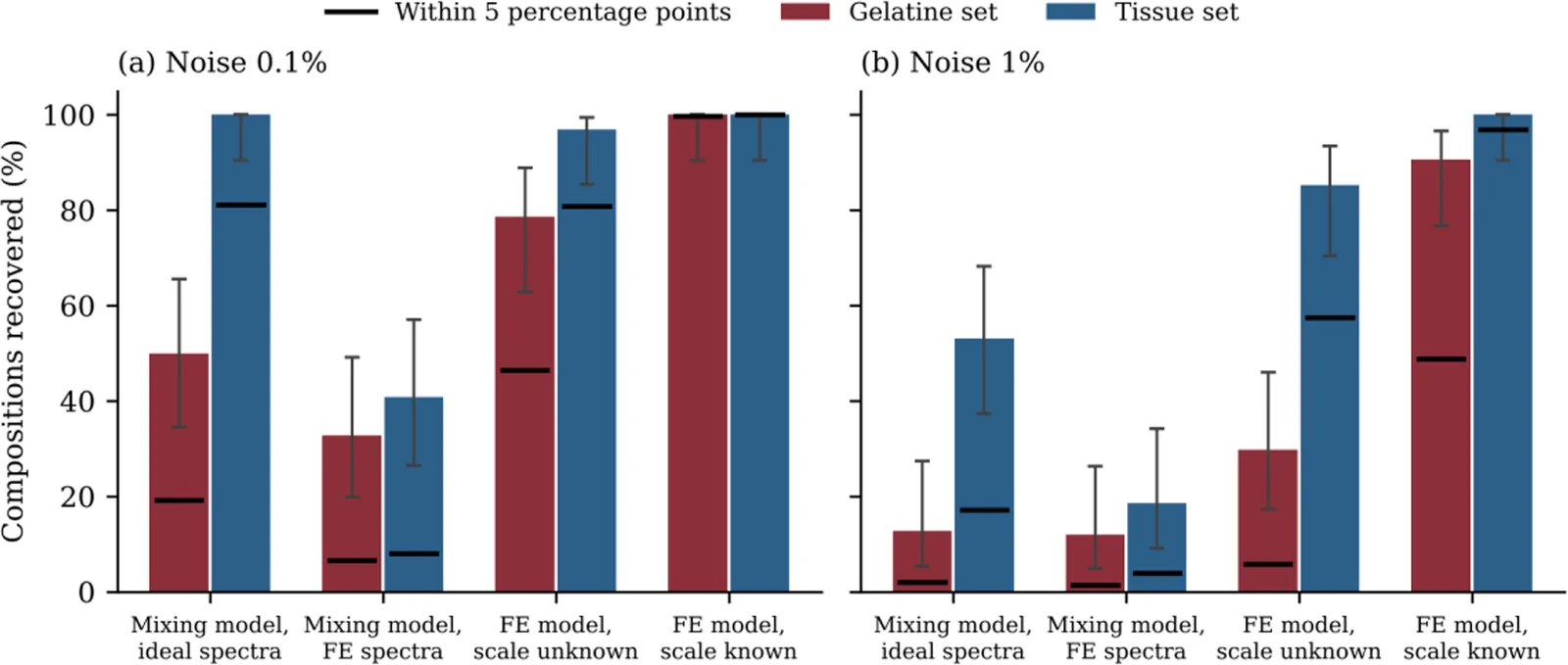
Recovery rate for the gelatine and tissue sets at (a) 0.1% noise and (b) 1% noise. The first group fits ideal spectra from the mixing model with the same model, and the other groups fit spectra from the FE model of spot electrodes. Bars show recovery within 15 percentage points with 95% confidence intervals, and black lines show recovery within 5 percentage points.

Table 5 gives the effect of errors that are the same at all frequencies. A phase error of ±0.1° reduced the recovery of the gelatine set with an unknown scale from 79% to 29%, while the tissue set remained at 98%. The uncorrected setup error of 120 pF and 14 ns prevented recovery of the gelatine set with an unknown scale. For the tissue set, it reduced the recovery with an unknown scale from 97% to 58%. A magnitude error of 17% reduced the recovery with a known scale to 69% for the gelatine set and 81% for the tissue set.

**Table 5:** Recovery rate in percent with errors that are the same at all frequencies. All spectra were fitted with the FE model and had random noise of 0.1%. A magnitude error is absorbed by a free scale factor, and it therefore has no effect when the scale is unknown.

**Added error**	**Gelatine, known scale**	**Gelatine, unknown scale**	**Tissue, known scale**	**Tissue, unknown scale**
None	100	79	100	97
Phase ±0.1°	100	29	100	98
Phase ±0.3°	47	10	100	94
Setup error 120 pF and 14 ns	27	0	100	58
Magnitude 17%	69	79	81	97

Table 6 gives the effect of a spread in tissue properties, with a phase error of ±0.1° in every case. With a known scale factor, recovery remained at 86% or more, and the typical errors stayed below 10 percentage points. With an unknown scale factor, recovery fell to 79% with a 5% spread, 74% with 10% and 62% with 20%, and the typical error in FM rose to 11–15 percentage points.

**Table 6:** Recovery of the tissue set when the true tissue properties differ from those used in the fit. All cases used the FE model, a phase error of ±0.1° and random noise of 0.1%. Typical errors are in percentage points.

**Spread in tissue properties**	**Known scale, recovered (%)**	**Known scale, typical error FM / FFM / TBW**	**Unknown scale, recovered (%)**	**Unknown scale, typical error FM / FFM / TBW**
None	100	1 / 2 / 1	98	5 / 3 / 3
5%	99	3 / 5 / 4	79	11 / 8 / 5
10%	97	3 / 6 / 6	74	12 / 9 / 7
20%	86	7 / 7 / 9	62	15 / 13 / 8

Table 7 gives the results when several realistic errors act together, namely a phase error of ±0.1°, a 10% spread in tissue properties and, for a known scale, a 17% magnitude error. Under these conditions, the tissue set recovered 86% of compositions with a known scale and 74% without it, with typical errors of 6–9 and 7–12 percentage points. With a 20% spread, recovery fell to 64% and 62%. The gelatine set recovered 67% and 29% of compositions under the same phase and magnitude errors.

**Table 7:** Recovery when several realistic errors act together. All cases used the FE model, a phase error of ±0.1° and random noise of 0.1%, and the 17% magnitude error applies only when the scale is known. Typical errors are in percentage points.

**Material set**	**Scale**	**Spread in tissue properties**	**Recovered (%)**	**Typical error FM / FFM / TBW**
Gelatine	Known, with 17% magnitude error	Not applicable	67	13 / 9 / 9
Gelatine	Unknown	Not applicable	29	32 / 16 / 17
Tissue	Known, with 17% magnitude error	10%	86	9 / 6 / 8
Tissue	Unknown	10%	74	12 / 9 / 7
Tissue	Known, with 17% magnitude error	20%	64	12 / 9 / 10
Tissue	Unknown	20%	62	15 / 13 / 8

Table 8 gives the recovery of the tissue set with the band extended beyond 251 kHz. The extension helped only when the tissue properties were known exactly. Up to 2 MHz, the error amplification factor fell from 61 to 38, and the typical error in FM for ideal spectra fell from 4.1 to 2.4 percentage points. With an unknown scale and a 10% spread in tissue properties, recovery was 74% over 1–251 kHz and 69–74% for the wider bands. With a 20% spread, it fell from 62% to 51% at 2 MHz. The uncorrected setup error reduced recovery to 23–47% for the wider bands, against 58% over 1–251 kHz.

**Table 8:** Recovery of the tissue set in percent with the band extended beyond 251 kHz. All cases used the FE model and random noise of 0.1%, unless stated otherwise. The first row gives the error amplification factor of the tissue set over each band.

**Test**	**1–251 kHz**	**1 kHz–500 kHz**	**1 kHz–1 MHz**	**1 kHz–2 MHz**
Error amplification factor	61	53	43	38
Unknown scale	97	98	99	98
Unknown scale, 1% noise	85	88	88	88
Unknown scale, phase ±0.1°, 10% spread	74	71	69	74
Unknown scale, phase ±0.1°, 20% spread	62	56	56	51
Known scale, phase ±0.1°, 17% magnitude, 10% spread	86	87	87	81
Known scale, phase ±0.1°, 17% magnitude, 20% spread	64	62	62	60
Unknown scale, uncorrected setup error of 120 pF and 14 ns	58	23	27	47

## Discussion

This work set out to identify what limits the recovery of three tissue volumes from a single surface spectrum. The measured phantoms provide the clearest answer. When the scale of the measurement was known, the fat fraction was recovered to within 1–3 percentage points. This held both for the two cylinders measured with spot electrodes and for the planar phantom measured with plates. The FFM and TBW fractions were considerably harder to separate. For the agar cylinder and the planar phantom, fits almost as good as the best were found over a wide range of TBW. This behaviour is characteristic of an ill-conditioned problem [26,27], in which the spectrum fixes FM but leaves the split between FFM and TBW open. Small, fixed differences between the model and the measurement can then move this split by several percentage points. For the gelatine set with a known scale, random noise of 0.1% alone would give a standard deviation of only 0.03 percentage points in FM. For FFM and TBW, it would give 0.3–0.4 percentage points. The errors of the measured phantoms were therefore dominated by such fixed differences rather than by noise.

The material spectra explain much of this behaviour. The FM and FFM materials of the gelatine set share the same gelatine base and differ mainly in salt content. This changes how well they conduct, but not the shape of their spectra. Without a known scale, the fit therefore had little to work with, and both gelatine phantoms were not recovered. The agar set brought the conductivity of FM much closer to that of real fat. Its FM spectrum also differed enough from the other two materials for the fit of the agar cylinder to fix the scale. Its error amplification factor was nevertheless higher than that of the gelatine set, because its FFM and TBW materials remained almost identical. The agar cylinder hence recovered FM without a known scale, while its FFM and TBW remained poorly determined. The spectra of real tissue were about 22 times less similar than those of the gelatine set. This arises mainly from the dispersion of muscle near 450 kHz, of blood near 1.2 MHz and of fat at low frequencies. An extension of the band to 2 MHz lowered the factor of the tissue set from 61 to 38. This change is small compared with the factor of 22 between the gelatine and tissue sets.

The simulations examined whether such tissue-like spectra could remove the necessity for a known scale. With exactly known tissue properties they could, and the typical errors were 3–5 percentage points. In practice, however, tissue properties are not known exactly. Kangasmaa et al. [39] found that the conductivity of fat in the forearm ranged from 0.12 to 0.31 S/m, and an earlier study of the leg found about 0.07 S/m [40]. With a spread of 10–20% in tissue properties and an unknown scale, the typical errors rose to 7–15 percentage points. A wider band did not change this result, since the dispersions that it resolves are those of the uncertain tissue properties. When several realistic errors acted together, the typical errors for the tissue set were 6–12 percentage points with a known scale and 7–15 percentage points without it (Table 7). These errors are similar in size to the individual error of about ±8 percentage points for current BIA [6–9]. Although this comparison is only indicative, since that figure refers to whole-body fat in people, it suggests that physics-based recovery is not yet clearly better than current practice.

The electrode model is a further source of error. Spot electrodes drive current through the outer layer, which was FM in these phantoms. Both the FE model and Ansys Maxwell showed this effect, with FM contributing 23% and 16% of the measurement against 9% for end plates. Similar effects of the electrode type and of surface layers have been reported in other studies [16,30,31,32]. For the measured cylinders, the FE model of the actual electrode positions predicted the impedance to within 1.1% and 6% with no fitted parameters, which supports this description of the current path. The two models, however, differed by 17% in absolute value. This difference lowered the recovery with a known scale to 69% for the gelatine set and 81% for the tissue set. Without a known scale, a fit of the Maxwell spectrum with the FE model overestimated FM by 16 percentage points for the gelatine set. This occurred even though the two models agreed closely in shape. When the material spectra are this similar, very small differences between models are sufficient to change the recovered composition. An electrode model validated against measurement is therefore required before a recovered composition can be trusted.

The measurement setup also had to be characterised before model and measurement could be compared in phase. The phase difference of the gelatine cylinder arose from the setup rather than from the phantom. Two precision resistors showed a capacitance of about 120 pF across the sample and a fixed delay of about 14 ns, both of which are common in such measurements [23,24,37]. The materials and the planar phantom were measured in a different cell, so their setup errors largely cancel in the mixing model, although the materials still carry them [38]. The agar cylinder agreed in phase without this correction, which suggests that the setup error depends on the connection and should be checked for each measurement. The planar phantom required a different correction, since each of its layers touches the electrodes and carries its own electrode polarisation. Its remaining difference below about 30 kHz is consistent with a change in polarisation between the material and phantom measurements, which depends on the state of the electrode surface [21]. One additional polarisation term reduced the difference from 0.57° to 0.19°, although this change was not measured directly. The correction of the materials also assumed the same polarisation for all three materials. A removal or rescaling of this term changed the recovered FM by less than 0.3 percentage points, although it moved the split between FFM and TBW. A four-electrode cell for the materials would remove the need for this correction.

Fig. 11 brings these findings together as two routes to recovery. In the first, the measured geometry fixes the scale, which then requires a validated electrode model and a corrected setup. In the second, the shapes of the material spectra fix the scale, which requires distinct spectra and well-known material properties. Table 9 lists each factor that limited recovery in this study, its observed effect and the corresponding requirement for a future test. The largest effects came from an unknown scale combined with similar material spectra, an uncorrected setup error and a spread in tissue properties. As such, a phantom intended to test physics-based recovery should use materials with clearly different spectra and a known geometry. Its setup should be checked against precision resistors, and the electrode model should be validated on the same phantom.

**Fig. 11:**
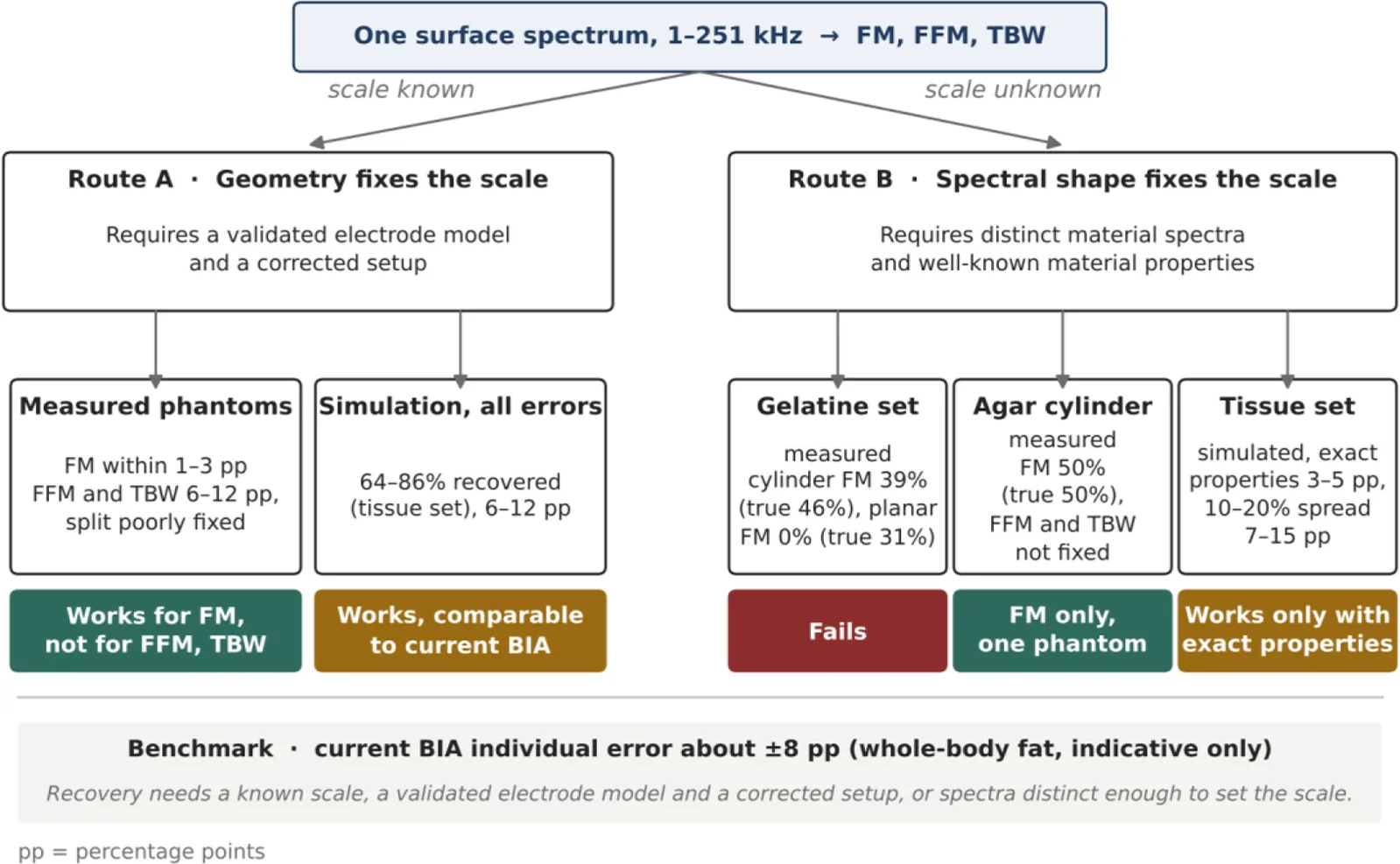
Two routes to recovering tissue volumes from one surface spectrum. In route A, the measured geometry fixes the scale. In route B, the shapes of the material spectra fix it instead. Values are taken from Tables 3, 6 and 7, and pp denotes percentage points. The agar result is based on a single phantom, and the benchmark of about ±8 percentage points refers to whole-body fat in people and is only indicative.

**Table 9:** Factors that limited recovery in this study and the requirement each places on a future test. Recovery rates are percentages of simulated compositions recovered within 15 percentage points with the FE model, unless stated otherwise.

**Factor**	**Effect observed**	**Requirement**
Similar material spectra	With an unknown scale and 1% noise, 30% were recovered for the gelatine set against 85% for the tissue set	Phantom materials with distinct spectral shapes
Unknown scale	Of the measured phantoms, only the agar cylinder gave FM, and its FFM and TBW were poorly determined	A measured geometry, or distinct spectra with well-known tissue properties
Constant phase error of ±0.3°	For the gelatine set with an unknown scale, recovery fell from 79% to 10%	Setup errors corrected to about 0.1°
Uncorrected setup error of 120 pF and 14 ns	For the tissue set with an unknown scale, recovery fell from 97% to 58%, and the gelatine set was not recovered	Setup error measured with precision resistors
Magnitude error of 17% from the electrode model	With a known scale, recovery fell from 100% to 69% for the gelatine set and from 100% to 81% for the tissue set	Electrode model validated on the same phantom
Spread of 10–20% in tissue properties	For the tissue set with an unknown scale, recovery fell from 98% to between 74% and 62%	Known scale or independent tissue properties

This study has several limitations. Only three compositions were tested physically, two with spot electrodes and one with plates, and the agar result in particular rests on a single phantom. Each phantom was built once, and the materials and phantoms were made in separate preparations of the same recipes. Test–retest measurements over several days were not made, and the temperature of the room was not logged. A common change in temperature alters the conductivity of all layers by about 2% per °C, and it therefore acts as a change in scale. The electrode positions of the cylinders were measured after the experiment, with an uncertainty of about 3 mm. The results for tissue-like materials come from simulation and remain predictions, and the FE model was used both to create the simulated spectra and to fit them.

The Maxwell comparison showed that a change of model can shift the recovered FM by 16 percentage points for materials with similar spectra. This test could not be repeated for the tissue set. Low-frequency tissue values, such as those of Gabriel et al. used here, are still debated [41]. These values also do not include the direction dependence of muscle, which is large in vivo below 10 kHz [39]. In addition, in vivo estimates of the conductivity of fat are several times higher than the value used here [39,40]. The phantom materials respond through interfaces within the gel, which differs from the cell membranes of real tissue. Finally, the 36 test compositions give confidence intervals of about ±15 percentage points on each simulated recovery rate. Notwithstanding these limitations, the combination of measured phantoms of known composition with a verified field model provides a quantitative account of where recovery succeeds and where it fails. Neither simulation nor measurement alone could provide this account.

Future work will follow three directions. The first is a set of phantoms with spot electrodes and tissue-like materials, which would test the simulated results directly. The second is a model of metal electrodes validated against such a phantom. The third is measurement over a wider frequency range, since the MFIA impedance analyser (Zurich Instruments, Zurich, Switzerland) extends beyond 1 MHz, where tissue spectra differ most. The simulations indicate, however, that a wider band helps only when the tissue properties are well known.

## Conclusion

This work set out to identify what limits the recovery of tissue volumes from a single surface bioimpedance spectrum. An FE model of the actual electrodes predicted two three-layer cylinders measured with spot electrodes to within 1.1% and 6% with no fitted parameters. With the scale known, the fat fraction of three phantoms was recovered to within 1–3 percentage points, whereas the fat-free and water fractions were poorly separated. Without a known scale, recovery failed for the gelatine phantoms, but the fat fraction of an agar phantom with a more distinct fat spectrum was still recovered. In simulations, tissue-like spectra removed the necessity for a known scale only when the tissue properties were known exactly, and with realistic errors acting together, the typical errors were 6–15 percentage points. Recovery therefore requires either a known scale, together with a validated electrode model and a corrected setup, or material spectra distinct enough to fix the scale. Future work will test these requirements in phantoms designed to meet them.
